# Extracellular Vesicles in Viral Infections: Two Sides of the Same Coin?

**DOI:** 10.3389/fcimb.2020.593170

**Published:** 2020-12-01

**Authors:** Sharon de Toledo Martins, Lysangela Ronalte Alves

**Affiliations:** ^1^ Gene Expression Regulation Laboratory, Carlos Chagas Institute, ICC-Fiocruz, Curitiba, Brazil; ^2^ Biological Sciences Sector, Federal University of Paraná (UFPR), Curitiba, Brazil

**Keywords:** extracellular vesicles, virus infection, host response, viral particles, immune response

## Abstract

Extracellular vesicles are small membrane structures containing proteins and nucleic acids that are gaining a lot of attention lately. They are produced by most cells and can be detected in several body fluids, having a huge potential in therapeutic and diagnostic approaches. EVs produced by infected cells usually have a molecular signature that is very distinct from healthy cells. For intracellular pathogens like viruses, EVs can have an even more complex function, since the viral biogenesis pathway can overlap with EV pathways in several ways, generating a continuum of particles, like naked virions, EVs containing infective viral genomes and quasi-enveloped viruses, besides the classical complete viral particles that are secreted to the extracellular space. Those particles can act in recipient cells in different ways. Besides being directly infective, they also can prime neighbor cells rendering them more susceptible to infection, block antiviral responses and deliver isolated viral molecules. On the other hand, they can trigger antiviral responses and cytokine secretion even in uninfected cells near the infection site, helping to fight the infection and protect other cells from the virus. This protective response can also backfire, when a massive inflammation facilitated by those EVs can be responsible for bad clinical outcomes. EVs can help or harm the antiviral response, and sometimes both mechanisms are observed in infections by the same virus. Since those pathways are intrinsically interlinked, understand the role of EVs during viral infections is crucial to comprehend viral mechanisms and respond better to emerging viral diseases.

## Extracellular Vesicles (EVs) in Viral Diseases

Extracellular vesicles (EVs) are membrane vesicles that have recently received considerable attention. EVs carry several RNA subtypes, proteins and DNA that can be functional in recipient cells after transfer. The smallest EV type, initially called an exosome (Johnstone et al., 1987), originates from multivesicular bodies, a part of the endocytic pathway. They were initially observed with a cup-shaped morphology through conventional transmission electron microscopy techniques, although nowadays it is known that this shape is a preparation artifact, and a round morphology is observed with more advanced cryo-EM techniques (Chuo et al., 2018). They are also enriched with molecules such as CD63, CD9 and CD81, Alix and TSG101; however, the distribution of these surface molecules varies greatly depending on the cell type, and no canonical markers applicable to all types of EVs have been described to date (Théry et al., 2018). Several other types of EVs have been identified, such as apoptotic bodies, microvesicles and vesicles specific to certain cell types, such as oncosomes secreted by tumor cells (Raposo and Stoorvogel, 2013). The exact biogenesis pathway of these different subtypes is not totally understood. Although EV pathways are active in homeostasis, they are very important under disease conditions. It was previously shown that the EV composition changes drastically during infection, especially infections by intracellular pathogens such as viruses, and host RNAs contained in EVs can affect viral recognition by the immune system to induce or restrict viral propagation in recipient cells (Yoshikawa et al., 2019).

EVs can affect recipient cells through different mechanisms. Cargo delivery by membrane fusion can transport functional molecules such as RNAs into recipient cells (Montecalvo et al., 2012). The endocytic uptake mechanisms can involve clathrin-dependent or clathrin-independent pathways, and heterogeneous populations of EVs are probably internalized by multiple mechanisms (Mulcahy et al., 2014). The clathrin-independent mechanisms can be mediated by caveolin or lipid rafts, and EVs can also be internalized by phagocytosis and micropinocytosis. Proteins and glycoproteins present on the surface of EVs and in recipient cells can also influence these mechanisms (Mulcahy et al., 2014). In addition to cargo delivery by direct fusion or internalization, EVs can also influence target cells through interaction with different receptors, such as lectins (Barrès et al., 2010), heparan sulfate proteoglycans (Christianson et al., 2013), conexins and integrins (Shimaoka et al., 2019). Extracellular vesicles can bind to the cell surface and remain attached to proteins like integrins or trigger intracellular signaling. They also can be internalized and directed to the endosomal pathway until they reach multivesicular endosomes (MVEs), where they can fuse with lysosomes directing their content to degradation and recycling (Tian et al., 2010). Vesicles near the MVE membrane can release their contents on the cytoplasm by back fusion escaping degradation (Bissig and Gruenberg, 2014), and this can also happen to vesicles attached directly to the plasma membrane. This process is important to deliver nucleic acids present on the EVs to the recipient cells, although is still not well understood (Van Niel et al., 2018). There is also evidence that some vesicles can be re-secreted by fusion of MVEs with the plasma membrane or through the early endocytic recycling pathway (Heusermann et al., 2016).

EVs are secreted by most cells, travel long distances within the body, and can be found in several bodily fluids, having great potential as diagnostic tools and in therapeutic and preventive interventions such as vaccine production (Dogrammatzis et al., 2020), as already shown for the influenza virus (Keshavarz et al., 2019), porcine respiratory reproductive syndrome virus (Montaner-Tarbes et al., 2019), and SARS coronavirus, in which they were able to induce the production of a high level of neutralizing antibodies (Kuate et al., 2007). For example, therapeutic EVs derived from mesenchymal stem cells have the ability to induce the differentiation of anti-inflammatory macrophages, inactivate T cells and induce regulatory immune cells such as T and B lymphocytes and dendritic cells. These vesicles can be used to treat acute inflammatory conditions such as severe cases of COVID-19 (Tsuchiya et al., 2020).

## Challenges to the Isolation and Detection of Extracellular Vesicles and Viral Particles

The methodologies used to isolate and characterize extracellular vesicles are very diverse, and each experimental model and scientific question poses its own challenges. Fortunately, efforts of researchers and scientific societies in the EV field are helping to identify better methods and standards to study EVs (Théry et al., 2018). Several established methods used for viral isolation, such as ultracentrifugation, precipitation with crowding reagents, cross-flow filtration, column chromatography and affinity purification, can also be used to isolate extracellular vesicles (McNamara and Dittmer, 2019). Although the ability to use these methods in both viral and EV fields is interesting, it poses difficulties for separating replicative viral particles from extracellular vesicles, especially because these vesicle/virus populations appear to exist on a continuum. Phenotypic characterization of those populations with antibodies, affinity purification after isolation and the use of strategies such as those involving viral replicons that do not secrete viral particles are potential research strategies. In addition, new techniques such as nanofacs and flow virometry seem promising in the search for better separation of these subpopulations (McNamara and Dittmer, 2019).

## Routes of Extracellular Vesicle Biogenesis—Exosomes and Microvesicles

Exosome biogenesis is very complex, can vary depending on the cargo, cell type and other stimuli received by the cell, with several mechanisms acting at the same time or sequentially (Edgar et al., 2014), generating an heterogeneous population of vesicles (Van Niel et al., 2018). Different sorting machineries can act on the same endosomal compartment (van Niel et al., 2011), or different machineries can target the same cargo, as observed for MHC class II (Buschow et al., 2009). For this reason, different subpopulations of EVs can coexist (Colombo et al., 2014). Viral infection can interfere with all cellular processes and the intervention with cellular metabolism and reorganization of internal membranes can end up crossing the pathways of EV biogenesis and viral budding, affecting early endosomal sorting machineries (Van Niel et al., 2018).

Exosomes are generated as intraluminal vesicles (ILVs) in the lumen of endosomes during their maturation to multivesicular endosomes (MVEs), involving several sorting mechanisms. They segregate content in membrane microdomains in the MVE membrane and generate smaller membrane vesicles by inward budding and fission (Van Niel et al., 2018). The ESCRT machinery was one of the first proteins to be discovered in this process (Hurley, 2008), acting in several steps, in which ESCRT-0 (also known as HRS) and ESCRT-1 gather ubiquitylated transmembrane cargos in microdomains, and ESCRT-II recruits ESCRT-III, responsible for fission and budding. Inactivation of the members of ESCRT family can affect the composition and release of vesicles (Colombo et al., 2013) and HRS seems to be required for exosome formation and secretion by dendritic cells (Tamai et al., 2010). Molecules like syntenin, ALIX and VPS32 are also important in this process (Baietti et al., 2012).

Exosomes can also be formed in an ESCRT independent manner. When the four ESCRT proteins are depleted, ILVs loaded with CD63 are still able to be formed (Stuffers et al., 2009). The first ESCRT independent pathway of exosome formation is mediated by neutral type II sphingomyelinase, that transforms sphingomyelin in ceramide (Trajkovic et al., 2008), allowing the formation of membrane subdomains (Goñi and Alonso, 2009) that create negative membrane curvatures. Ceramide can also be transformed in sphingosine-1-phosphate and activate a receptor that is crucial for cargo sorting (Kajimoto et al., 2013). Tetraspanins like CD81, CD83, CD9 and CD63 can also regulate biogenesis in an ESCRT-independent way, since they can form clusters and induce budding in membrane microdomains with tetraspanins and other transmembane and cytosolic proteins (Charrin et al., 2014). CD63 was also shown to be involved in endosomal sorting (van Niel et al., 2011; van Niel et al., 2015), cargo targeting and biogenesis of exosomes. CD81 presents a cone-like structure that can accommodate cholesterol inside it, and their clustering can induce inward budding. Tetraspanins can also regulate the intracellular route of cargo like integrins (Odintsova et al., 2013). The type of cargo can also affect the sorting on exosomes. Transmembrane cargos are heavily depending on endosomal machineries, and the affinity of molecules like GPI anchored proteins to lipid rafts could affect membrane properties and be involved in budding (De Gassart et al., 2003). Soluble proteins can be sequestered inside ILVs by co-sorting with chaperones (HSP70, HSC70) found in exosomes of different origins (Géminard et al., 2004). Also, proteins with certain modifications like ubiquitination or farnesylation are enriched in ILVs, but the mechanisms are still unknown. The sorting of nucleic acids is differential, since some types of miRNA motifs are preferentially sorted inside ILVs (Villarroya-Beltri et al., 2013), but passive loading can also occur. Machineries involved in nucleic acid sorting to EVs include the ESCR-II subcomplex that can have RNA-binding properties (Irion and St Johnston, 2007), sequestration of RBPs in membrane domains (Perez-Hernandez et al., 2013) or the presence of RNA silencing complexes like miRNA induced silencing complex (miRISC), argonaute 2 (AGO2), KRAS-MEK signaling, major vault protein and Y box binding protein (YBX1) (Gibbings et al., 2009; McKenzie et al., 2016; Shurtleff et al., 2016; Teng et al., 2017).

Although apoptotic bodies are known for a long time, the mechanisms of microvesicle release from the membrane of healthy cells are starting to be uncovered only recently. Rearrangements in the plasma membrane (lipid components, proteins, and CA+2 levels) are important in this process (Johnstone et al., 1987). Aminophospholipid translocases (flippases and floppases), scramblases and calpain are dependent on Ca2+ and rearrange membrane phospholipids, bending the membrane and restructuring the actin cytoskeleton, favoring membrane budding and microvesicle formation (Piccin et al., 2007). Defects in the scramblase can impair the exposure of phosphatidylserine and the production of platelet-derived procoagulant microvesicles (Piccin et al., 2007). Other lipids like cholesterol can also contribute to microvesicle biogenesis, since their depletion impair the formation of microvesicles in neutrophils (Del Conde et al., 2005). Cytoskeleton regulators that alter actin dynamics, like RHO GTPases and RHO-associated protein kinase (ROCK), can induce microvesicle biogenesis (Li et al., 2012). Metabolic changes can also affect their release, as seen for the Warburg effect, when the inhibition of glutaminase activity dependent of RHO GTPases can block microvesicle biogenesis (Wilson et al., 2013). For the cargo selection, lipids and other cargos with membrane affinity can localize to lipid raft membrane domains, as happen to oligomeric cytoplasmic proteins that are anchored in plasma membrane (Yang and Gould, 2013), and cytosolic components need to bind to the inner leaflet of the plasma membrane. This mechanism is very similar to the budding of HIV and retroviruses (Van Niel et al., 2018). The mechanisms of nucleic acid targeting to the cell membranes is still unknown, but is still unclear how nucleic acids, which are generally found in microvesicles, are targeted to the cell surface. The presence of zip code RNA sequence motifs in the 3´-UTR regions of mRNA can be one of the possible targeting mechanisms to microvesicles (Bolukbasi et al., 2012).

## Viruses use Intracellular Membranes to Evade the Immune Response and Complete Their Cycle

Viruses can exploit intracellular membranes to complete their cycles and propagate, creating structures called replicative organelles (Wolff et al., 2020) and using cellular secretion mechanisms to facilitate particle formation and budding. Positive sense RNA viruses, such as nidoviruses (Angelini et al., 2014), arteriviruses (Knoops et al., 2012), flaviviruses (Gosert et al., 2002), coronaviruses (Snijder et al., 2006; Ulasli et al., 2010), have an interesting mechanism of replication involving internal membrane rearrangements in host cells, generating double-membrane structures known as replicative organelles (den Boon and Ahlquist, 2010). These structures contribute to immune evasion by hiding viral components from the immune system and working as scaffolds that anchor viral replication and transcription complexes (V’kovski et al., 2015). This membrane reorganization can be induced by viral proteins, as shown for SARS-CoV, that can induce membrane disorder and proliferation (through nsp3, in both full length and truncated forms, and nsp6), membrane pairing (with the synergic action of nsp3 and nsp4) and induction of perinuclear vesicles around the microtubule organizing center (through nsp6) (Angelini et al., 2013). The result of these rearrangements is demonstrated by the formation of double-membrane vesicles and convoluted membranes connected to the rough endoplasmic reticulum (Ulasli et al., 2010). Components of ER-Golgi cellular trafficking were also shown to be involved in the formation of these structures (Reggiori et al., 2010), and they are also involved in EV formation, being a possible point of overlap to allow the presence of viral components inside EVs. The degree of induction of intracellular membrane structures can vary between coronavirus strains, although it is not necessarily correlated with pathogenicity (Maier et al., 2016). This mechanism also indicates that infection changes cellular lipid metabolism and that some enzymes involved in lipid processing are crucial for the formation of these membrane structures. The inhibition of cytosolic phospholipase A2a significantly reduces the formation of coronavirus particles *in vitro*, suggesting that the formation of these internal membrane structures is essential for completion of the viral replication cycle (Müller et al., 2017).

## Vesicles or Viral Particles? Overlap Between Viral Budding and EV Biogenesis

In addition to secreting replicative viral particles, infected cells can also secrete other structures containing viral proteins and nucleic acids that can activate the immune system or impact recipient cells, favoring viral propagation (van der Grein et al., 2018). There is ongoing discussion about the classification of these particles, since they can be either host EVs containing viral molecules or defective viral particles. It is difficult to isolate pure populations of these different types of vesicles since they are of similar size, density and composition, and most isolation methods cannot be used to separate them (van der Grein et al., 2018). The replicative viral structures found inside host EVs can be complete viral particles or “quasi-enveloped” viruses (viruses that are classically nonenveloped but can be found “cloaked” inside host EVs) (Feng et al., 2014).

The virology field classifies some viruses as enveloped when their capsids are surrounded by host membrane; these viruses usually bud directly from the plasma membrane or through an exocytic pathway without necessarily promoting cell death. Examples of enveloped viruses are HIV, influenza, dengue and SARS-Cov2. Nonenveloped viruses, such as hepatitis A virus (HAV), coxsackievirus, norovirus, poliovirus and rhinovirus, typically promote cell lysis, which is required for their release, and are not surrounded by host membrane (Lindenbach, 2013; Altan-Bonnet, 2016). Nevertheless, in 2013, the distinction between enveloped and nonenveloped viruses became less clear when both types of particles were found *in vivo* and in the extracellular medium of liver cells infected in HAV (Feng et al., 2013). Cellular analysis was used to track them inside multivesicular bodies (MVBs) to their cells of origin, and the depletion of ESCRT proteins blocked their release, suggesting an overlap of viral particle release and the exosome biogenesis pathway (Altan-Bonnet, 2016).

Viral particle formation pathways sometimes include the endosomal machinery that produces EVs. Usually, viruses enter cells through endocytosis (although some enveloped viruses can fuse directly to cell membranes) and release their nucleic acids, which are undergoing replication/transcription, and new virions can bud through the cell membrane or are released after cell lysis (Urbanelli et al., 2019). In 2003, the “Trojan Horse” hypothesis was formulated by Gould and colleagues who showed that EVs secreted by dendritic cells infected with HIV were able to infect T CD4+ lymphocytes (Gould et al., 2003). Gag, an HIV structural protein, is known to directly recruit Alix and ESCRT-1 proteins (Votteler and Sundquist, 2013), important components of exosome formation machinery. The expression of coronavirus E and M proteins is sufficient to generate virus-like particles even in the absence of the other viral components (Maeda et al., 1999). Additionally, some herpesviruses can interact with the ESCRT machinery during the formation of the viral envelope in endosomal compartments and the trans Golgi network (TGN) (Sadeghipour and Mathias, 2017). For this reason, some nonenveloped viruses can be found in “quasi-enveloped” states inside exosomes when they are released, cloaked in host membranes and lack viral surface proteins, as observed for the hepatitis A virus (HAV) and hepatitis E (Nagashima et al., 2014). Recent research has demonstrated that collective viral spread involving viral aggregates can favor viruses and promote the evolution of defective interfering particles, and extracellular vesicles may also have a role in this process (Andreu-Moreno and Sanjuán, 2020), since aggregation can change the internalization route of EVs, favoring phagocytosis and micropinocytosis (Feng et al., 2010).

**Figure 1:**
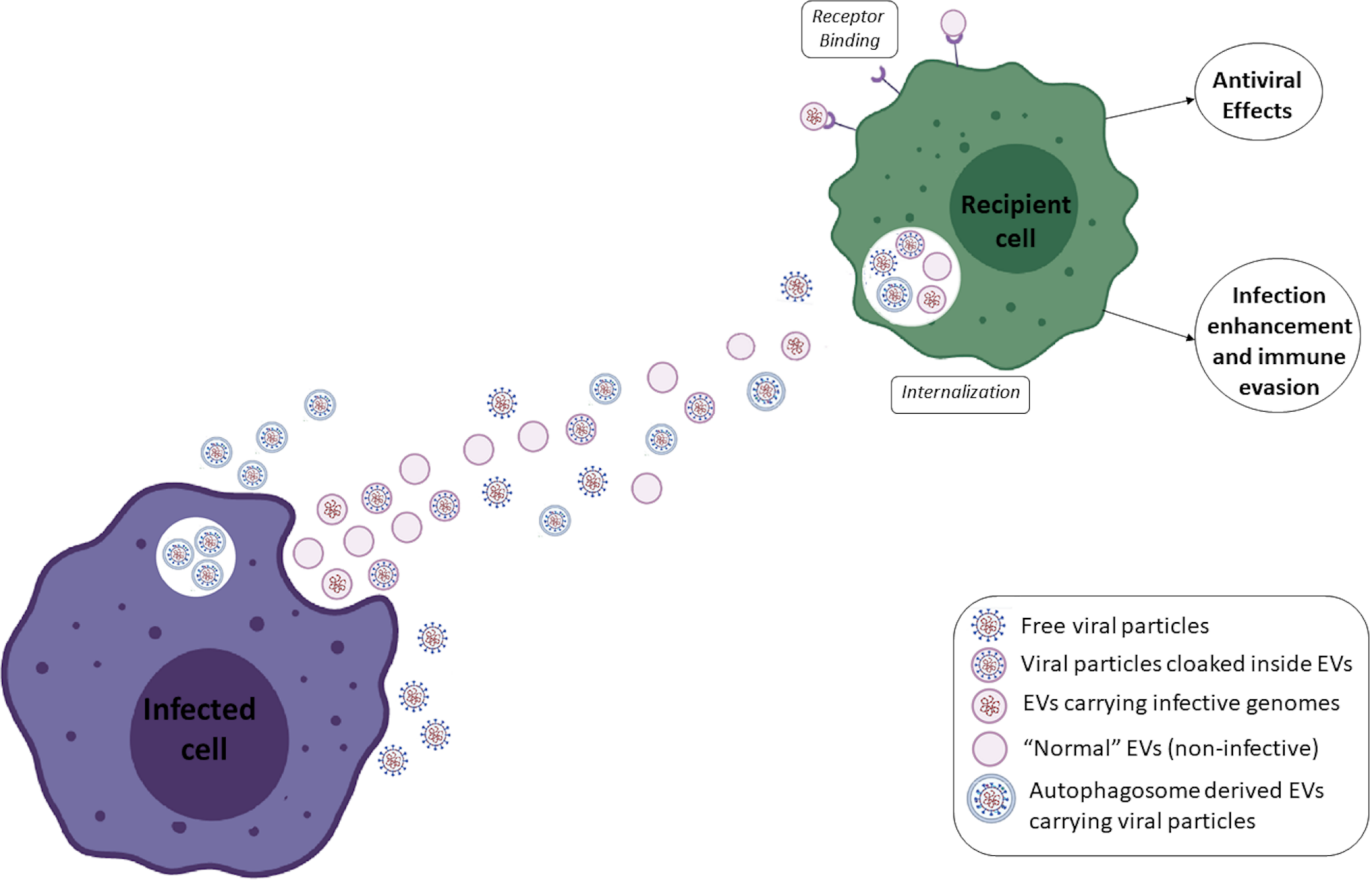
Scheme depicting an interplay between extracellular vesicles, viruses and subpopulations of viral particles.

Even when envelopes are acquired in canonical pathways, the exosomal pathway can be exploited by viruses to facilitate its own transmission, as seen for porcine reproductive and respiratory syndrome virus (PRRSV) (Wang et al., 2018), herpes simplex virus HSV-1 (Bello-Morales and López-Guerrero, 2020), enterovirus 71 (Gu et al., 2020), and RVFV, which pack viral RNA and proteins inside vesicles (Ahsan et al., 2016); HIV, which facilitates macrophage infection through EVs (Kadiu et al., 2012); HBV, which can directly induce replication through EVs of infected cells (Li et al., 2019); and HTLV-1, which exports functional viral proteins inside EVs to uninfected cells (Jaworski et al., 2014). Cells infected with rhinovirus secrete EVs that induce the upregulation of viral receptors in monocytes, which allows the virus to infect alternative cell types (Miura, 2019). Mosquito cells infected with DENV secrete larger EVs than uninfected cells, and these structures contain virus-like particles that are able to infect other cells (Reyes-Ruiz et al., 2019). Quiescent CD4+ T lymphocytes are usually refractory to HIV1 infection; however, EVs from infected cells can make them permissive to viral replication through the action of ADAM17 and Nef (Arenaccio et al., 2014). Additionally, Nef-containing EVs can modulate lipid rafts in recipient cells, facilitating the fusion of new viral particles with these cells and increasing infection (Dubrovsky et al., 2020). For HCV, it was shown that replication-competent sub genomic RNAs can be transferred through EVs and establish infection in recipient cells, even when complete viral particles are absent (Longatti et al., 2015). Thus, HCV infectivity was independent of classical HCV receptors or viral envelope proteins, rendering them partially resistant to antibody neutralization, which may be an immune evasion strategy used by several other viruses (Ramakrishnaiah et al., 2013). Vesicles containing genomic RNAs found in HCV patients also carried Ago2, HSP90 and miR-122, which facilitate viral stability and replication (Bukong et al., 2014; Altan-Bonnet, 2016). It was also shown that ZIKV is able to induce the amplification of EV production through increased expression and activity of SMPD3 and that EVs containing viral RNAs and proteins promote viral transmission (Zhou W. et al., 2019). FMDV (foot and mouth disease virus) can also be transmitted through host EVs that carry genomic RNAs and some viral proteins, and FMDV replication is not fully blocked by neutralizing antibodies, suggesting an immune evasion mechanism (Zhang et al., 2019). For SFTS (a tick-borne bunyavirus associated with hemorrhagic fever), exosomes from infected cells contained viable virions that were able to infect cells by an alternative route independent of classical receptors (Silvas et al., 2016). They enter cells by endocytosis, similar to naked virions, but they are uncoated, and their genome is released by different pathways. Viruses that usually have lytic cycles can also be released from cells in a nonlytic way (Rivera-Serrano et al., 2019). A scheme depicting an interplay between extracellular vesicles, viruses and subpopulations of viral particles can be observed in **Figure 1**.

## Secretory Autophagy and Viral Budding

Some nonenveloped viruses can exit the cell in vesicles that originate from autophagosomes instead of MVBs, as shown for rhinovirus, poliovirus and coxsackievirus (Bird et al., 2014; Robinson et al., 2014). Autophagosomes usually have a double membrane and are formed from the endoplasmic reticulum, mitochondria or plasma membrane (Cuervo, 2010), and through their natural degradation mechanism, they process large quantities of cytoplasm to provide nutrients during stress (Feng et al., 2015). However, instead of fusing with lysosomes, they can fuse with the plasma membrane to release vesicles containing viral particles. This “secretory autophagy” is observed in uninfected cells (Ponpuak et al., 2015) from which useful molecules and organelles are released into the extracellular space (Altan-Bonnet, 2016). These vesicles are enriched in phosphatidylserine, which can be important for infection (Chen et al., 2015), as observed for classical enveloped viruses such as vaccinia, dengue, Ebola and pseudotype lentivirus (Amara and Mercer, 2015). The viruses inside exosomes or large autophagosome-derived vesicles were found to be more infective than the viruses released when the autophagosomes were lysed (Altan-Bonnet, 2016). It is believed that these vesicles can be disrupted once internalized by host cells in acidic compartments such as acidified endosomes, releasing the viral particle (Bird et al., 2014). A recent study reviewed the interaction of MERS, SARS-Cov, and SARS-CoV2 on autophagic processes. The literature data are very inconsistent regarding the role of autophagy in coronavirus replication, with some studies suggesting that it is necessary, while others stating that replication is autophagy-independent, and some studies showing that the virus can inhibit the autophagy process. This ambiguity may indicate a nonclassical pathway of autophagy that may be related to the secretory form of autophagy described above (Yang & Shen, 2020).

## EVs in Immune Communication and Cytokine Responses During Infection

The infection process can drastically change the composition of host EVs, changing the proportions of host proteins and RNAs inside these structures (Hoen et al., 2016). During infection, EVs can amplify inflammation and deflagrate antiviral responses (Urbanelli et al., 2019) and can also mediate communication between immune cells and other cell types (Isola and Chen, 2017). The involvement of EVs in viral infection and/or host interactions in disease has already been described for several viruses, such as rabies (Wang et al., 2019b), coronaviruses (Maeda et al., 1999; Kuate et al., 2007; Börger et al., 2020; Deffune et al., 2020; Hassanpour et al., 2020; Inal, 2020a; Inal, 2020b; Kumar et al., 2020; O’Driscoll, 2020; Tsuchiya et al., 2020; Urciuoli and Peruzzi, 2020), HCV (Bartosch et al., 2003; Timpe et al., 2008; Dreux et al., 2012; Bukong et al., 2014), HBV (Jia et al., 2017; Li et al., 2019), HIV (Princen et al., 2004; Khatua et al., 2009; Xu et al., 2009; Lenassi et al., 2010; Bernard et al., 2014; Raymond et al., 2016; Sampey et al., 2016; Kodidela et al., 2018; Haque et al., 2020; Ranjit et al., 2020), HPV (Honegger et al., 2015; Guenat et al., 2017; Sadri Nahand et al., 2019; Chiantore et al., 2020), HSV (Temme et al., 2010; Han et al., 2016; Deschamps and Kalamvoki, 2018) dengue (Martins et al., 2018; Mishra et al., 2019; Sung et al., 2019), HTLV-1 (Pinto et al., 2019), Zika (Zhou W. et al., 2019; Martínez-Rojas et al., 2020), West Nile (Slonchak et al., 2019), Epstein Baar (Keryer-Bibens et al., 2006; Klibi et al., 2009; Zhao M. et al., 2019), influenza (Liu Y. et al., 2019; Maemura et al., 2020), and SFTS (Silvas et al., 2016).

EV secretion occurs in several body systems during homeostasis, and it represents an important communication pathway in the immune system (Isola and Chen, 2017). Vesicles transferred between immune cells can transmit signals that trigger an increase or decrease in cytokine production and transfer antigens, and some EVs are able to trigger direct antigen presentation (Lindenbergh and Stoorvogel, 2018). EVs carry cytokines and cytokine-related RNAs that can elicit the production of target molecules in recipient cells, having a role in the antiviral response (Urbanelli et al., 2019). EVs secreted by infected cells are able to activate other cells, as observed when vesicles secreted from U937 macrophages infected with DENV-2 activate endothelial cells (Velandia-Romero et al., 2020). Infection with West Nile virus changes the composition of host microRNAs, small noncoding RNAs and mRNAs in EVs, and the enriched RNAs are related to viral processing and host responses to infection (Slonchak et al., 2019). It was also observed that two strains of dengue virus with different virulence profiles induce the secretion of EVs with drastically different RNA compositions from monocyte-derived dendritic cells (Martins et al., 2018). When taken up by macrophages, vesicles from HIV-infected cells containing Nef can trigger the inflammasome, inducing the secretion of proinflammatory cytokines (Mukhamedova et al., 2019). EVs released by airway epithelial cells infected with RSV (respiratory syncytial virus) have increased expression of regulatory small RNAs and can stimulate chemokine production in monocytes without transferring infective particles (Chahar et al., 2018).

Sometimes high levels of proinflammatory cytokine production can contribute to disease severity, as seen for several infectious diseases, and EVs can mediate this process. EVs isolated from bronchoalveolar fluid of mice infected with a highly pathogenic avian influenza virus (H5N1) showed enrichment with miR-483-3p, which stimulates innate immune responses in pneumocytes (Maemura et al., 2018). This molecule was also enriched in the serum of infected mice, and pneumocyte-derived EVs enriched with this molecule increased the expression of proinflammatory cytokine genes in vascular endothelial cells, suggesting the involvement of EVs in the inflammatory pathogenesis of H5N1 (Maemura et al., 2020). A similar process occurs for dengue hemorrhagic fever, a severe disease in which massive secretion of cytokines and high vascular hyperpermeability can lead to shock syndrome, and extracellular vesicles were shown to be involved in this process (Mishra et al., 2019). A summary of the main findings associated to the EVs and viruses are described in **Table 1**.

**Table 1:** Summary of the main findings associated to the EVs and viral infections.

EVS FAVORING VIRAL PROPAGATION	
Mechanism	Virus
EVs facilitate viral transmission	HSV-1 (Bello-Morales and López-Guerrero, 2020), KSHV (Chen et al., 2020)., NDV (Zhou C. et al., 2019), PRRSV (Wang et al., 2017),
	enterovirus 71 (Gu et al., 2020), HCV (Bukong et al., 2014), HIV (Kadiu et al., 2012), SFTS (Silvas et al., 2016)
Viral RNAs/proteins inside EVs	Coronavirus (Maeda et al., 1999), EBV (Keryer-Bibens et al., 2006), HCV (Kouwaki et al., 2017)., HTLV-1, (Jaworski et al., 2014), RVFV (Ahsan et al., 2016), ZIKV (Zhou W. et al., 2019; Martínez-Rojas et al., 2020)
Infectious virus-like particles/cloaked virions inside EVs	DENV (Reyes-Ruiz et al., 2019), enterovirus 71 (Gu et al., 2020), HCV (Bartosch et al., 2003; Timpe et al., 2008)
Transfer of infective RNA through EVs withouth complete viral particles	HCV (Longatti et al., 2015), FMDV (Zhang et al., 2019),
EVs turn cells more permissive to infection, membrane/receptor modulation	HIV (Arenaccio et al., 2014; Dubrovsky et al., 2020), Rhinovirus (Miura, 2019)
Host molecules in EVs facilitate viral stability and replication in recipient cells	HBV (Li et al., 2019), HCV (Bukong et al., 2014; Altan-Bonnet, 2016), HIV (Arenaccio et al., 2014; Ranjit et al., 2020)
Amplification of EV production	ZIKV (Zhou W. et al., 2019)
EVs from uninfected cells can activate latent viruses	HIV (Barclay et al., 2020)
**EVS RELATED TO IMMUNE RESPONSES**	
**Mechanism**	**Virus**
EVs from infected cells are able to activate other cells	DENV (Velandia-Romero et al., 2020; Mishra et al., 2019)
RNAs inside EVs related to host responses to infection	DENV (Martins et al., 2018a), H5N1 (Maemura et al., 2018; Maemura et al., 2020), HBV (Zhao X. et al., 2019), HIV (Bernard et al., 2014), HSV-1 (Han et al., 2016; Huang et al., 2019), influenza (Liu Y. et al., 2019), Rabies (Wang et al., 2019a), RSV (Chahar et al., 2018), West Nile (Slonchak et al., 2019)
EVs from infected cells can trigger the secretion of proinflammatory molecules in other cells	HIV (Sampey et al., 2016; Mukhamedova et al., 2019), H5N1 (Maemura et al., 2018; Maemura et al., 2020), HBV (Zhao X. et al., 2019), RSV (Chahar et al., 2018)
EVs involved in IFN-mediated responses	DENV (Martins et al., 2018), HBV (Yao et al., 2019; Zhao X. et al., 2019), HCV (Dreux et al., 2012; Okamoto et al., 2014), HIV-1 (Khatua et al., 2009), HSV-1 (Huang et al., 2019), influenza (Liu et al., 2019)
EVs that can restrict viral replication	Rabies (Wang et al., 2019a), HBV (Zhao X. et al., 2019), HIV (Ouattara et al., 2018),
Induction of massive inflammatory responses/vascular permeability	DENV (Sung et al., 2019)
EVs can block/impair viral propagation	Enterovirus (Chen et al., 2015), Influenza (Liu Y. et al., 2019), HIV-1 (Khatua et al., 2009), HSV-1 (Han et al., 2016; Deschamps and Kalamvoki, 2018; Huang et al., 2019), Rabies (Wang et al., 2019a)
EVs can induce antibody production	SARS (Kuate et al., 2007)
**EVS CAN HELP VIRUSES BLOCK ANTIVIRAL RESPONSES**	
**Mechanism**	**Virus**
EVs reduce IFN-mediated antiviral protection in recipient cells	enterovirus 71 (Wang et al., 2018), HBV (Shi et al., 2019), HCV (Florentin et al., 2012)
EVs carry host RNAs related to antiviral response blocking	enterovirus 71 (Wang et al., 2018)., NDV (Zhou C. et al., 2019)
More cytopatic effect in recipient cells	NDV (Zhou C. et al., 2019)
EVs turn recipient cells more permissive to infection	rhinovirus (Zhou et al., 2017).
EVs impair other antiviral mechanisms/promote immune evasion	EBV (Klibi et al., 2009), HCV (Ashraf Malik et al., 2019), HIV (Schaefer et al., 2008; Xu et al., 2009; Lenassi et al., 2010; De Carvalho et al., 2014), KSHV (McNamara et al., 2019)
**EVS SECRETED DURING INFECTION CAN TRIGGER SECONDARY DISEASES**	
**Mechanism**	**Virus**
Oncogenig effect	gamma-herpes virus (Zheng et al., 2019), human papillomavirus (Honegger et al., 2015; Ambrosio et al., 2019; Chiantore et al., 2020), HIV (Sharma, 2019), MVP (Teng et al., 2017)
Accumulation of beta amyloid plaques	HIV (Fulop et al., 2019).
Trigger inflammation	human papillomavirus (Sadri Nahand et al., 2019)
Contribute to tissue fybrosis	HCV (Kim et al., 2019).
Mediate chemoresistance	HBV (Liu D. et al., 2019)
Mediate autoimmunity/transplant rejection	respiratory viruses (Gunasekaran et al., 2020).
EVs involved in viral latency/persistant infections	HIV (Olivetta et al., 2019; Barclay et al., 2020), HCV (Ashraf Malik et al., 2019),
Thrombosis induction	SARS CoV-2 (Inal, 2020a), Nomura et al., 2020

## EVs Can Elicit and Propagate Antiviral Responses

The protective effect of EVs during infection can also involve classical antiviral pathways, such as the interferon response, because effector molecules, such as interferon stimulated genes (ISGs), can be carried to other cells (Li et al., 2013). The secretion of type I interferon is a potent and conserved antiviral response strategy. IFN protein is produced and then secreted into the extracellular space after pathogen-associated molecular patterns (PAMPs) are recognized by Toll-like receptors. After secretion, the produced IFN molecules can bind to surface receptors in other cells and trigger a protective response (Schneider et al., 2014). The translocation of NFKb to the nucleus induces the transcription of several ISGs, which are the true antiviral effectors of these pathways. The EV pathway is linked to the IFN response in several ways. First, viral components from infected cells can be transferred to other cells through EVs, where they will induce IFN production. An example of this is what happens with plasmacytoid dendritic cells (pDCs), that have an important role in innate immunity by recognizing viral nucleic acids through TLR7 and TLR9 (Gilliet et al., 2008), inducing their activation and production of IFN among other molecules. When transferred to pDCs, EVs from infected cells can be internalized and their viral RNA can activate TLR7 (Assil et al., 2015). The activation of IFN response in pDCs by extracellular vesicles may be more powerful than the one induced by only mature virions, since EVs from HCV infected cells can induce a strong IFN response in pDCs (Dreux et al., 2012), while conventional HCV particles may block TLR7 induced signaling as an immune evasion strategy (Florentin et al., 2012). A recently explored area gaining attention involves the transfer of ISGs through EVs. The mRNAs for ISGs can be transferred to bystander cells or over long distances, and the recipient cells can translate these mRNAs (Li et al., 2013). Complete ISG proteins can also be transferred (Borghesan et al., 2019; Yao et al., 2019). The viral entry machinery can also be used by the cells to transfer antiviral protection since the same receptors that some viruses use to bind the cells can also bind EVs. This is exemplified by macrophage-derived exosomes that depend on T-cell immunoglobulin and mucin receptor 1 (TIM-1), a receptor used by Hepatitis A Virus (HAV) (Yao et al., 2018). EVs secreted by THP-1 macrophages treated with IFN-alpha are enriched with proteins related to the “defense response to virus” and “type I IFN signaling pathway”. Some of the proteins for ISGs found in this work were upregulated both in macrophages treated with IFN and in the EVs secreted by them (IFI44L, IFIT1, ISG15, EIF2AK2, MX2, IFIT3, MX1, STAT2, OAS3, IFI16, OAS2, STAT1 and IFIT2), while one ISG was found upregulated only in EVs (SAMHD1) (Yao et al., 2019). These vesicles also present potent antiviral activity when delivered to hepatocytes infected with hepatitis B, suggesting that effector antiviral molecules induced by IFN can be transferred through exosomes (Yao et al., 2019).

The presence of HBV-miR-3 in EVs secreted by HBV-infected cells can induce macrophage polarization to an M1 phenotype, increase IFN production, activate the Jak/STAT signaling pathway and induce IL-6 secretion. These actions may restrict HBV replication and suppress the acute liver cell injury caused by HBV (Zhao X. et al., 2019). Hepatocytes infected with HCV can produce EVs loaded with infective HCV RNA that, when internalized by pDCs, can trigger type I IFN production upon TLR7 binding. This effect is attenuated when ESCRT-I and ESCRT-III are depleted from the infected hepatocytes, suggesting a correlation with the EV pathway (Dreux et al., 2012). Similarly, EVs secreted by cells with HCV replicons induced the TLR3-mediated production of IFN I and III by delivering viral RNAs to DCs (Okamoto et al., 2014) (Kouwaki et al., 2017).

Influenza-infected cells secrete EVs containing miR-1975 that induce interferon expression in recipient cells (Liu Y. et al., 2019). Cells infected with herpes simplex virus (HSV-1) produce EVs packed with miR-H28 and miR-H29, which are able to restrict viral transmission to uninfected cells through the induction of IFN-gamma production (Huang et al., 2019). Other mechanisms of EV-mediated viral inhibition can also occur, as observed for MRC-5 cells infected with rabies, that show increased production of EVs containing miR-423-5p, which inhibits RABV replication in neighboring recipient cells (Wang et al., 2019a). Exosomes from the microenvironment and biofluids can also modulate viral infection, as shown for seminal EVs, which seems to have a protective effect against HIV infection. They can directly inhibit HIV-1 cellular entry, prevent transmission of HIV from vaginal epithelial cells to monocytes, T lymphocytes and PBMCs. They can also inhibit replication after internalization by blocking reverse transcriptase activity and inhibiting binding of transcription factors to the HIV1 promoter (Ouattara et al., 2018). This helps to explain the low infection rates of people exposed to the virus (Ouattara et al., 2018; Welch et al., 2019).

## Viruses Can Exploit EV Machinery to Block Antiviral Responses and Allow Their Own Propagation

EVs have an important role in transferring antiviral molecules, facilitating propagation of responses such as interferon activity. However, several viruses are known to disrupt the defense pathway mediated by IFN, thus evading the immune response (**Table 1**). For example, EVs loaded with IFITM2 delivered to dendritic cells reduce the capacity of the recipient cells to produce IFN-alpha and thus counteract HBV infection (Shi et al., 2019). EVs secreted from cells infected with NDV carry miR-1273f, miR-1184 and miR-198, which are able to block IFN-beta antiviral responses and increase the virus-induced cytopathic effect in recipient cells (Zhou C. et al., 2019). Similarly, EVs secreted by human epithelial cells infected with enterovirus 71 (EV71) can transfer miR-30a to macrophages to target the MyD88 gene, suppressing type I IFN production (Wang et al., 2018).

Viruses can also use the EV pathway to make neighboring cells more permissive to infection. Monocytes treated with conditioned media from rhinovirus-infected epithelial cells exhibited increased secretion of proinflammatory cytokines and ICAM1, which makes the monocytes more permissive to infection and viral replication (Zhou et al., 2017). Usually, TCD4+ T cells are able to secrete EVs with surface CD4 molecules. These receptors act as decoys, binding the HIV1 virus and inhibiting the infection of new cells. However, in addition to reducing CD4 on cell surfaces, the HIV protein Nef can also reduce the expression of CD4 on EVs, blocking this mechanism to allow viral propagation (De Carvalho et al., 2014). When transferred to B cells through EVs, the HIV Nef protein can impair the production of IgG and IgA antibodies (Xu et al., 2009). Nef can induce cell death when delivered to bystander TCD4+ cells (Lenassi et al., 2010) and the degradation of the viral receptors CD4 and MHC-1 (an important molecule that presents viral antigens to the immune system) through the action of Nef-interacting protein B-cop (Schaefer et al., 2008). In addition, EVs secreted by uninfected cells can activate the transcription of latent viruses in HIV-1-infected cells through cellular SRC-1 and the PI3K/AKT/mTOR pathway (Barclay et al., 2020).

## EVs Secreted During Viral Infections Can Cause Secondary Disease

EVs secreted during the course of a viral infection can have effects in many parts of the body, triggering secondary diseases (**Table 1**). For example, gamma-herpes virus can induce an oncogenic effect (Zheng et al., 2019), among several others. There is also evidence that EVs contribute to the beta-amyloid plaque accumulation that occurs in the brain of HIV patients, probably contributing to cognitive decline (Fulop et al., 2019). EVs secreted during human papillomavirus infection can carry miRNAs that induce cervical inflammation (Sadri Nahand et al., 2019) and are related to the development of squamous cell carcinoma (Ambrosio et al., 2019). EVs secreted by HCV-infected hepatocytes contain miR-192 and, when transferred to hepatic stellate cells (HSCs), induce TGF-β1 upregulation, triggering differentiation into myofibroblasts. This process contributes to the liver fibrosis induced by HCV (Kim et al., 2019). HIV-1-infected cells secrete EVs containing TAR RNAs that have pro-growth and pro-survival effects on cancer cells, having the potential to induce tumor progression and malignancy (Sharma, 2019). Kaposi’s sarcoma-associated herpesvirus (KSHV), a tumor-associated virus, can induce the proliferation, migration and transcriptional changes of uninfected endothelial cells through EVs, thereby evading the pathogen recognition surveillance system (McNamara et al., 2019). It was also shown that salivary EVs from HIV patients carried *tar*, *tat* and *nef* RNAs but not TAT or Nef proteins. Treatment with these EVs increased the KSHV infection rate of oral epithelial cells through the EFG receptor (EGFR), and this effect was blocked by cetuximab, a drug that targets EGFR. This facilitation of KSHV infection caused by HIV EVs can explain the high rates of Kaposi sarcoma in HIV patients and indicates that this virus can break the epithelial barrier to spread through the body (Chen et al., 2020). HBV-associated liver cancer presents more chemoresistance than non-HBV tumors. EVs from HBV-infected cancer cells were able to downregulate the apoptosis of recipient cells upon drug treatment, modulate cell death through the CMA pathway and upregulate Lamp2A, suggesting that the EVs induced by infection can mediate chemoresistance through chaperone-mediated autophagy (Liu D. et al., 2019). Patients with respiratory viral infections after lung transplantation had circulating EVs containing lung self-antigens, the 20S proteasome and viral antigens that can trigger a rejection response against the transplanted lung and lead to allograft dysfunction (Gunasekaran et al., 2020). EVs in this context can also facilitate the establishment of persistent viral infections. HIV can persist in latent reservoirs that are not recognized by the immune system, leading to a rebound infection after discontinuation of antiretroviral therapy, and it is believed that EVs are crucial for the preservation of these reservoirs (Olivetta et al., 2019). For HCV, CD81+ EVs loaded with viral particles allow the virus to escape immune surveillance, helping to establish persistent infections (Ashraf Malik et al., 2019). Additionally, systemic hemorrhagic diseases that involve vascular permeability, such as dengue hemorrhagic fever, show the involvement of EVs during pathogenesis. Besides being important in immune cell communication, like EVs secreted by mdDCs that carry RNAs related to antiviral response and inflammatory cytokines (Martins et al., 2018), DENV infection can induce the secretion of platelet EVs that cause massive inflammatory responses by activation of CLEC5A and TLR2 on macrophages and neutrophils (Sung et al., 2019), and induce the formation of Neutrophil Extracellular Traps (Mishra et al., 2019). Additionally, it was already discussed that extracellular vesicles can be involved in thrombosis events observed after infection by several types of viruses (Nomura et al., 2020).

## Concluding Remarks

Despite experimental difficulties, the field of extracellular vesicles in viral infections is growing and has tremendous potential to solve healthcare problems. The EVs can carry infective viral particles, they also influence the response of surrounding cells and turning them more susceptible to infection. On the contrary the EVs can also help the host cell to fight the infection, by triggering antiviral responses and cytokine secretion. As stated, the EVs can either facilitate or impair the antiviral response, and sometimes both mechanisms are observed in infections by the same virus. Since those pathways are intrinsically interlinked, understand the role of EVs during viral infections is crucial to comprehend viral mechanisms and respond better to emerging viral diseases. In summary, several mechanisms of virus and EV biogenesis are shared, and knowledge in one field can help to advance the prospects of the other. Understanding the interplay between viruses and extracellular vesicles can also help to develop mechanisms to respond better to public health threats caused by viral pathogens.

## Author Contributions

SM and LA wrote and corrected the manuscript. All authors contributed to the article and approved the submitted version.

## Funding

This work received financial support from Inova Fiocruz/Fundação Oswaldo Cruz [Grant number VPPCB-07-FIO-18-2-52] and CNPq [Grant number 442317/2019-0]. LA is a research fellow awardee from CNPq.

## Conflict of Interest

The authors declare that the research was conducted in the absence of any commercial or financial relationships that could be construed as a potential conflict of interest.
